# A Multilevel Investigation of Conduct Problems in Adolescents: Insights from Neural, Cognitive, and Environmental Perspectives

**DOI:** 10.1192/j.eurpsy.2025.1165

**Published:** 2025-08-26

**Authors:** C. Mou, Y. Shi, P. Liu, Y. Wang

**Affiliations:** 1 School of Psychology, University of Nottingham, Nottingham, United Kingdom; 2 Department of Psychology, National University of Singapore, Singapore, Singapore; 3 State Key Laboratory of Cognitive Neuroscience and Learning, Beijing Normal University, Beijing, China

## Abstract

**Introduction:**

Conduct problems (CP) in adolescents are associated not only with long-term personality and social development challenges, but also impose significant burdens on families, schools, and communities.

**Objectives:**

While numerous risk factors for CP have been identified in prior research, a comprehensive understanding of the underlying deficit mechanisms remains incomplete.

**Methods:**

Utilizing data from the Adolescent Brain Cognitive Development (ABCD) study (N = 11,875), the largest longitudinal investigation of brain development and child health in the United States, we conducted a systematic analysis of the neural, cognitive, and environmental features linked to CP. The findings were further tested for generalizability across diverse cross-cultural datasets.

**Results:**

Our results propose a novel framework that accounts for cognitive deficits associated with CP, while also highlighting the interactions between biological and environmental factors in the development and potential remission of CP in adolescents.

**Conclusions:**

These insights provide valuable directions for future research and intervention strategies targeting adolescent conduct problems.

**Disclosure of Interest:**

None Declared

